# Influencing Factors of Mental Health Status of Dentists Under COVID-19 Epidemic

**DOI:** 10.3389/fpsyt.2022.933514

**Published:** 2022-07-11

**Authors:** Junping Li, Jiaoyang Guo, Juan Zhao, Yan Guo, Cheng Chen

**Affiliations:** ^1^Key Laboratory of Shaanxi Province for Craniofacial Precision Medicine Research, College of Stomatology, Xi’an Jiaotong University, Xi’an, China; ^2^Laboratory Center of Stomatology, College of Stomatology, Xi’an Jiantong University, Xi’an, China; ^3^Department of Emergency, College of Stomatology, Xi’an Jiantong University, Xi’an, China; ^4^Department of General Dentistry, College of Stomatology, Xi’an Jiantong University, Xi’an, China

**Keywords:** COVID-19, depression, anxiety, stress, perceived wellness survey, dentists

## Abstract

**Objective:**

To investigate dentists’ psychological status and influencing factors in Shaanxi Province during the COVID-19 epidemic and assess their perceived wellness.

**Methods:**

The study was conducted among dentists from Shaanxi Province in China. The basic information was collected through the network questionnaire star platform. Depression, Anxiety, and Stress Scales (DASS-42) and Perceived Wellness Survey (PWS) were used to assess subjects’ psychological status and perceived wellness. Univariate linear regression analysis and multivariate analysis were performed on the influencing factors of depression, anxiety, and stress, and *t*-test and analysis of variance were used to analyze the perceived wellness results.

**Results:**

The results demonstrated that 33.2% of the surveyed dentists were in a state of depression, 37.1% were anxious, and 34.4% reported stress among 256 subjects. Linear Regression analysis results showed that: “years of working,” “the impact of COVID-19 on their life, work, and sleep,” “worrying about occupational exposure/virus infection,” “lacking the awareness of prevention and control measures,” “overtime work during the epidemic,” “worrying about participating in the supporting work,” and “continuous exhaustion from work” were significant contributors to depression, anxiety, and stress status. In addition, the results of PWS found that each dimension of PWS was correlated with depression, anxiety, and stress state, which indicates the individual’s physical and mental health state was associated with multiple factors.

**Conclusion:**

COVID-19 has significantly impacted dentists’ mental health in Shaanxi Province. With these findings, we aim to educate and promote targeted interventions that can be utilized to improve dentists’ mental health by analyzing the influencing factors.

## Introduction

COVID-19 has rapidly and globally spread since the first case was reported in Wuhan, China, in December 2019. The World Health Organization announced on 30 January 2020 that the COVID-19 outbreak was listed as an internationally concerned public health emergency (1). By the time the author wrote this article, there were 500,186,525 confirmed COVID-19 cases and 6,190,349 deaths reported to WHO, and a total of 11,294,502,059 vaccine doses had been administered (2).

The global outbreak of COVID-19, individual fear of infectious disease, quarantine policies for infected and close contact people, the global economic recession, and unemployment have negatively affected many people’s mental health (3). Many reports show that the stress caused by COVID-19 has had a range of adverse effects on their health, such as insomnia, anxiety, depression, and the exacerbation of chronic diseases (4). Furthermore, with the increasing number of cases, medical workers suffered from great psychological and work pressure and faced a high risk of infection during the epidemic.

The novel COVID-19 is highly contagious. Respiratory droplets and close contact are the main routes of transmission. The concept of COVID-19 “aerosol” transmission was first introduced in China’s “Diagnosis and Treatment Protocol (Trial Fifth Edition).” “Aerosol transmission in a relatively confined environment” is now confirmed as one of the routes of transmission of COVID-19 (5). Oral treatment and surgery usually produce a large number of aerosols and droplets. Oral environments include face-to-face contact with patients, long-term exposure to saliva, blood, other body fluids, airborne microorganisms, and the use of sharp instruments that increase the risk of infection (6). The COVID-19 virus can survive in aerosols for more than 3 h, and its attachment to some surfaces can be detected even after 72 h (7). Patients must have masks removed for oral diagnosis and treatment, leading to the inability to maintain a safe interpersonal distance between dentists and their patients. In addition, the dentist is also exposed to the patient’s saliva, blood, and other close contact exposures, which increases the risk of infection (8). Some scholars have found that oral epithelial cells contain a large number of Angiotensin-Converting Enzyme 2 (ACE2), which has been proved to be an important receptor for the COVID-19 virus (9), and the expression of ACE2 in small salivary glands is significantly greater than that in the lung (10). Based on these characteristics, dentists face a high risk of infection in their work, affecting their mental state and concerns of exposure.

During the outbreak stage, the stomatology departments of some general hospitals and private dental clinics in Shaanxi Province stopped receiving treatment. Instead, the stomatological hospitals provided treatment with the principle of “one patient, one room, one doctor, one care and one disinfection.” Predictably, dentists facing this high risk of exposure and firm quarantine policies can harm their physical and mental health.

Scholars who surveyed dentists from 30 different countries found that more than 2/3 (78%) of dentists were anxious and worried about the impact of COVID-19 (11). At present, there are many studies on the mental health status of frontline medical staff during the epidemic period at home and abroad. However, according to our literature review, there are few studies on the mental health status of dentists during the COVID-19 epidemic period in China. This article used different scales to analyze the mental health status of dentists in Shaanxi Province. We hope that through our research, we can find out the possible influencing factors which affect the mental health of dentists during the COVID-19 epidemic, and provide targeted psychological counseling for dentists.

## Data and Methods

### Participants

This study was conducted in Shaanxi Province in China between 17 January 2022 and 23 January 2022. All questionnaires were distributed through an online questionnaire survey platform. All participants were informed of the principles for filling out the form and signed an online informed consent form before answering. The inclusion criteria were designated as (1) on duty during the outbreak; (2) can skillfully use smartphones to fill out questionnaires. Exclusion criteria were designated as (1) had psychological problems before the study (such as depression, anxiety, insomnia, etc.); (2) non-dental medical staff; (3) cannot use smartphones. This study has been approved by the Medical Ethics Committee of Xi’an Jiaotong University, Hospital of Stomatology (Approval No.xjkqll [2022]NO.001). The researchers kept all questionnaire data confidential.

### Survey Tools

The tool used is called questionnaire star,^1^ which were anonymous online questionnaires. To ensure the validity of the data, each IP can only be answered once.

### Questionnaires

#### General Demographic Information

We obtained general demographic information (including gender, age, marital and fertility status, education level, years of working, nature of the hospital, etc.) about the respondents through the self-designed questionnaire.

#### Depression, Anxiety, and Stress Scales

The depression, anxiety, and stress scales (DASS-42) is a self-report tool with 42 items designed to identify the distinction symptoms between anxiety and depression and reveal their common feature called stress (12). The questionnaire has been translated into multiple languages and has proven cross-cultural validity (13). The DASS-42 scale classifies depression, anxiety, and stress into five grades: normal, mild, moderate, severe, and very severe (scoring criteria: depression — normal: 0–9; mild: 10–13; moderate: 14–20; severe: 21–27; very severe: 28 + . Anxiety — normal: 0–7; mild: 8–9; moderate: 10–14; severe: 15–19; very severe: 20 + . Stress — normal: 0–14; mild: 15–18; moderate: 19–25; severe: 26–33; very severe: 34 +). A higher score indicates a higher depression, anxiety, and stress level. The details are shown in Table 1. The Cronbach’s alpha reliability factor for the whole scale was 0.968 and reliability factors of 0.930, 0.894, and 0.923 for depression, anxiety, and stress (Cronbach’s alpha obtained from this sample). It is worth noting that the results do not provide any clinical diagnostic significance. If they were concerned about their psychological condition, they were instructed to seek a professional doctor in time.

**TABLE 1:** Depression, anxiety, and stress scales (DASS-42) scoring criteria.

	Depression	Anxiety	Stress
Normal	0–9	0–7	0–14
Mild	10–13	8–9	15–18
Moderate	14–20	10–14	19–25
Severe	21–27	15–19	26–33
Very severe	27+	20+	34+

#### Perceived Wellness Survey

Perceived wellness survey (PWS) is a valid scale for researching and evaluating interventions in the field of perceived health, with good reliability and validity (14, 15). The PWS contains 36 items which included six dimensions: physical (1, 7, 13, 19, 25, 31), emotional (2, 8, 14, 20, 26, 32), social (3, 9, 15, 21, 27, 33), psychological (4, 10, 16, 22, 28, 34), spiritual (5, 11, 17, 23, 29, 35), and intellectual (6, 12, 18, 24, 30, 36) which were selected based on the strength of theoretical support and the quality of empirical evidence supporting each. The PWS assesses the perceived health of individuals and their physical, mental, and spiritual states and the social health they exhibit through relationships with others. Therefore, it is used as a comprehensive questionnaire to investigate an individual’s overall health (16, 17). For the positive questions, scores of 6, 5, 4, 3, 2, and 1 represent “completely agree,” “agree,” “somewhat agree,” “somewhat disagree,” “disagree,” and “completely disagree.” The negative questions were scored contrariwise. A higher score indicates a better perception of wellness. The Cronbach’s alpha reliability factor for the whole scale was 0.911 and 0.703, 0.675, 0.649, 0.643, 0.665, and 0.743 for physical, emotional, social, psychological, spiritual, and intellectual, respectively (Cronbach’s alpha obtained from this sample).

### Date Analysis

SPSS 26.0 software was used for statistical analysis. Counting data is expressed in frequency, percentage, mean, and standard deviation.

Univariate linear regression was used for univariate analysis to explore the factors that might impact subjects’ depression, anxiety, and stress status during the COVID-19 pandemic. The statistically significant variables in the univariate analysis were taken as independent variables, and the scores of the three subscales in DASS-42 were taken as the dummy variables. The stepwise regression analysis was used for multi-factor analysis. In this study, the severe and very severe groups were combined into one group, *p* < 0.05 indicated a statistical difference.

The *t*-test and chi-square test were used to compare the PWS scores of subjects with different demographic characteristics. The relationship between six dimensions scores of PWS and depression, anxiety, and stress states were analyzed by bivariate correlation analysis, *p* < 0.05 indicated statistical difference.

## Results

### General Demographic Data

A total of 256 questionnaires were collected in this study, of which 256 were valid, with an effective rate of 100%. Basic information on subjects, such as gender, age, marital and fertility status, whether or not they live alone, general health, education level, years of working, and professional title, were collected (Table 2).

**TABLE 2:** General demographic characteristics of participants.

Characteristic	n	%
**Gender**		
Male	77	30.1
Female	179	69.9
**Age**		
21–30	108	42.2
31–40	123	48.0
41–50	17	6.6
≥51	8	3.1
**Live alone**		
Yes	80	31.3
No	176	68.7
**Marital status**		
Married	167	65.2
Unmarried	89	34.8
**Fertility status**		
None	110	43.0
1 Child	110	43.0
≥2 Children	36	14.0
**Past medical history**		
Yes	20	7.8
No	236	92.2
**Level of education**		
College or below	22	8.6
Undergraduate	136	53.1
Postgraduate and above	98	38.3
**Years of working**		
Under 5 years	119	46.5
6–10 years	87	34.0
More than 11 years	50	19.5
**Professional titles**		
Junior	143	55.9
Intermediate	96	37.5
Senior	17	6.6

### Characteristics of Depression, Anxiety, and Stress Status of Dentists During the COVID-19 Epidemic

Among the 256 questionnaires collected, 85 cases (33.2%) showed depression, 95 cases (37.1%) showed anxiety, and 88 cases (34.4%) showed stress. The results showed statistically significant differences in depression, anxiety, and stress in the general health status (*p* < 0.05). There was a statistical difference in anxiety states among dentists in different medical institutions (*p* < 0.05). Likewise, there were statistically significant differences in stress states between different educational levels and different working years (*p* < 0.05), as shown in Table 3.

**TABLE 3:** Comparison of depression, anxiety, and stress among dentists with different demographic characteristics.

Demography feature	Depression (*n* = 85, 33.2%)			χ^2^	*p*	Anxiety (*n* = 95, 37.1%)			χ^2^	*p*	Pressure (*n* = 88, 34.4%)			χ^2^	*p*
	Mild	Moderate	Severe			Mild	Moderate	Severe			Mild	Moderate	Severe		
**Gender**				4.31	0.23				1.22	0.748				0.58	0.901
Male	7.8%	9.1%	11.7%			3.9%	15.6%	14.3%			11.7%	14.3%	7.8%		
Female	11.7%	16.2%	7.3%			7.3%	16.8%	14.5%			14.0%	11.7%	8.9%		
**Age**				13.46	0.14				9.04	0.434				19.69	0.200
21–30	7.4%	8.3%	7.4%			5.6%	12.0%	11.1%			7.4%	8.3%	5.6%		
31–40	13.8%	18.7%	8.1%			7.3%	20.3%	15.4%			17.9%	17.1%	9.8%		
41–50	11.8%	17.6%	11.8%			0%	17.6%	23.5%			11.8%	5.9%	11.8%		
≥51	0%	12.5%	25.0%			12.5%	12.5%	25.0%			25%	12.5%	25%		
**Live alone**				1.07	0.79				1.04	0.792				1.94	0.585
Yes	8.8%	12.5%	7.5%			7.5%	18.8%	12.5%			13.8%	12.5%	5.0%		
No	11.4%	14.8%	9.1%			5.7%	15.3%	15.3%			13.1%	12.5%	10.2%		
**Marital status**				6.35	0.096				2.05	0.562				6.67	0.083
Unmarried	5.6%	11.2%	6.7%			4.5%	14.6%	12.4%			9.0%	11.2%	4.5%		
Married	13.2%	15.6%	9.6%			7.2%	17.4%	15.6%			15.6%	13.2%	10.8%		
**Fertility status**				7.07	0.315				7.59	0.269				11.34	0.079
None	6.8%	12.8%	6.8%			5.1%	13.7%	11.1%			10.3%	10.3%	5.1%		
1 Child	15.2%	16.2%	9.5%			6.7%	21.0%	15.2%			16.2%	17.1%	10.5%		
≥2 Children	8.8%	11.8%	11.8%			8.8%	11.8%	23.5%			14.7%	5.9%	14.7%		
**Past medical history^1^**				19.79	0.00*				12.99	0.005*				18.12	0.00*
Yes	5.0%	15.0%	35.0%			10.0%	15.0%	40.0%			25.0%	15.0%	30.0%		
No	11.0%	14.0%	6.4%			5.9%	16.5%	12.3%			12.3%	12.3%	6.8%		
**Level of education**				10.67	0.099				10.85	0.093				21.93	0.001*
College or below	22.7%	22.7%	13.6%			13.6%	27.3%	18.2%			18.2%	36.4%	13.6%		
Undergraduate	9.6%	16.2%	8.8%			6.6%	19.1%	14.7%			14.0%	14.0%	8.8%		
Postgraduate and above	9.2%	9.2%	7.1%			4.1%	10.2%	13.3%			11.2%	5.1%	7.1%		
**Years of working**				7.97	0.240				11.89	0.065				17.58	0.007*
Under 5 years	9.2%	10.1%	6.7%			5.0%	10.9%	10.9%			9.2%	8.4%	5.9%		
6–10 years	13.8%	14.9%	9.2%			6.9%	23.0%	14.9%			19.5%	16.1%	6.9%		
More than 11 years	8.0%	22.0%	12.0%			8.0%	18.0%	22.0%			12.0%	16.0%	18.0%		
**Professional title**				12.03	0.061				5.28	0.508				9.43	0.151
Junior	10.5%	11.9%	9.8%			6.3%	18.2%	11.9%			11.2%	11.9%	7.7%		
Intermediate	10.4%	19.8%	4.2%			6.3%	16.7%	16.7%			15.6%	15.6%	7.3%		
Senior	11.8%	0%	23.5%			5.9%	0%	23.5%			17.6%	0%	23.5%		
**Medical institution**				7.11	0.311				14.09	0.029*				11.55	0.073
Public specialized hospitals	8.3%	10.3%	8.3%			5.5%	12.4%	11.0%			11.7%	9.0%	6.2%		
Pubic general hospitals	12.7%	20.0%	9.1%			10.9%	16.4%	20.0%			18.2%	12.7%	10.9%		
Private dental clinics	14.3%	17.9%	8.9%			3.6%	26.8%	17.9%			12.5%	21.4%	12.5%		

*^1^The “Past medical history” means heart diseases, hypertension, diabetes, hepatitis, nephritis and other systemic diseases.*

*p < 0.05.

### Univariate Linear Regression Analysis of Depression, Anxiety, and Stress Status Among Dentists During the COVID-19 Epidemic

The factors that may affect dentists’ depression, anxiety, and stress state during the epidemic period were taken as independent variables. The total depression, anxiety, and stress scores in the DASS-42 scale were taken as dependent variables to conduct a one-way linear regression analysis. The results show that the influencing factors: “years of working,” “the influence degree of life and work, “the influence degree of sleeping,” “lacking cognition of prevention,” “worry about occupational exposure/virus infection,” “overtime work during the epidemic,” “water and electricity supply, transportation security,” “worried about participating in supporting,” “stress has nowhere to go, and the need for psychological counseling cannot be met,” and “continuous exhaustion from work” is positively correlated with depression, anxiety, and stress (*p* < 0.05). “General health status” was inversely related to depression, anxiety, and stress (*p* < 0.05), “marital status” was positively correlated with depression and stress (*p* < 0.05), “level of education” was negatively associated with depression and stress (*p* < 0.05), “lacking prevention and control materials” was positively correlated with anxiety and stress (*p* < 0.05), and “no guarantee of income” was positively associated with depression (*p* < 0.05). Furthermore, “fertility status,” “participation in outbreak-related work,” “guilt for not being able to take care of family,” and “fear of infection from colleagues” were positively associated with stress (*p* < 0.05). See Table 4 for details.

**TABLE 4:** Univariate linear regression analysis of the status of depression, anxiety, and/or stress among dentists during the COVID-19 epidemic.

Variables	Depression (DASS-42)		Anxiety (DASS-42)		Stress (DASS-42)	
	β (95%CI)	*P*	β (95%CI)	*P*	β (95%CI)	*P*
Marital status	0.099 (0.420,3.818)	<0.01	0.069 (−0.801,2.868)	0.268	0.146 (0.435, 4.836)	<0.05
Fertility status	0.086 (−0.438,2.464)	0.171	0.119 (−0.038,2.460)	0.057	0.177 (0.679, 3.675)	<0.01
General health status	−0.383 (−4.874,−2.638)	<0.01	−0.381 (−4.187,−2.254)	<0.01	−0.429 (−5.533, −3.248)	<0.01
Level of education	−0.126 (−3.311,−0.50)	<0.05	−0.107 (−2.644,0.178)	0.087	−0.209 (−4.581, 1.222)	<0.01
Years of working	0.138 (0.170,2.770)	<0.05	0.168 (0.423,2.657)	<0.01	0.206 (0.947, 3.630)	<0.01
Participation in outbreak-related work	0.064 (1.164,3.671)	0.308	0.105 (−0.298,3.862)	0.093	0.145 (0.457, 5.466)	<0.05
The influence degree of life and work	0.140 (0.188,2.866)	<0.05	0.160 (0.364,2.669)	<0.01	0.177 (0.634, 3.415)	<0.01
The influence degree of sleeping	0.163 (0.323,2.230)	<0.01	0.154 (0.213,1.863)	<0.05	0.190 (0.561, 2.544)	<0.01
Lacking of cognition of prevention	0.172 (0.770,4.502)	<0.01	0.195 (0.974,4.183)	<0.01	0.168 (0.743, 4.645)	<0.01
Worried about occupational exposure/virus infection	0.148 (0.383,3.985)	<0.05	0.161 (0.497,3.601)	<0.01	0.229 (1.677,5.381)	<0.01
Lacking of prevention and control materials	0.121 (0.018,3.172)	0.053	0.141 (0.215,2.963)	<0.05	0.137 (0.205,3.531)	<0.05
Overtime work during the epidemic	0.154 (0.309,2.693)	<0.05	0.166 (0.373,2.427)	<0.01	0.193 (0.736,3.209)	<0.01
Water and electricity supply, transportation security	0.171 (0.853,5.049)	<0.01	0.126 (0.054,3.702)	<0.05	0.162 (0.715,5.106)	<0.01
Noguarantee of income	0.136 (0.230,4.398)	<0.05	0.087 (−0.528,3.091)	0.164	0.089 (0.605,3.772)	0.155
Worried about participating in supporting	0.136 (0.355,6.602)	<0.05	0.145 (0.498,5.885)	<0.05	0.148 (0.699,7.214)	<0.05
Stress has nowhere to go, and the need for psychological counseling cannot be met	0.324 (3.693,7.861)	<0.01	0.279 (2.468,6.122)	<0.01	0.280 (3.009,7.428)	<0.01
Continuous work of exhaustion	0.220 (1.675,5.745)	<0.01	0.234 (1.654,5.157)	<0.01	0.226 (1.865,6.111)	<0.01
Guilt for not being able to take care of family	0.096 (−0.448,3.594)	0.126	0.097 (−0.364,3.126)	0.120	0.144 (0.372,4.571)	<0.05
Fear of infection from colleagues	0.076 (−0.841,3.585)	0.223	0.108 (−0.224,3.587)	0.083	0.151 (0.539,5.124)	<0.05

### Multivariate Analysis of Depression, Anxiety, and Stress Status Among Dentists During the COVID-19 Epidemic

Based on the one-way linear analysis, multiple linear regression analysis was performed based on the statistically significant variables in the DASS-42 scale and the dependent variables for the total scores of depression, anxiety, and stress. The results showed that the depression status was positively correlated with “years of working,” “no guarantee of income,” “stress has nowhere to go, and the need for psychological counseling cannot be met,” and negatively correlated with “general health status” (*R*^2^ = 0.262, *p* < 0.05). The anxiety status was positively correlated with “years of working,” “stress has nowhere to go, and the need for psychological counseling cannot be met,” “lack of prevention and control materials,” and was negatively correlated with “general health status” (*R*^2^ = 0.243, *p* < 0.05). The stress status was positively correlated with “years of working,” “level of education,” “worried about occupational exposure/viral infection,” “overtime work during the outbreak,” “stress has nowhere to go, and the need for psychological counseling cannot be met,” and was negatively correlated with “general health status” (*R*^2^ = 0.332, *p* < 0.05). The details are shown in Tables 5–7.

**TABLE 5:** Multiple linear regression analysis of dentists’ depression status during the COVID-19 epidemic.

Variable	Depression (DASS-42 score)	
	β (95% CI)	*p*
General health status	−0.332 (−4.319, −2.190)	0.000
Years of working	0.138 (0.300, 2.623)	0.014
No guarantee of income	0.127 (0.328, 3.984)	0.021
Stress has nowhere to go, and the need for psychological counseling cannot be met	0.303 (3.472, 7.334)	0.000

**TABLE 6:** Multiple linear regression analysis of dentists’ anxiety status during the COVID-19 epidemic.

Variable	Anxiety (DASS-42 score)	
	β (95%CI)	*p*
General health status	−0.332 (−3.661, −1.793)	0.000
Years of working	0.136 (0.229, 2.264)	0.017
Lacking of prevention and control materials	0.130 (0.277, 3.179)	0.020
Stress has nowhere to go, and the need for psychological counseling cannot be met	0.259 (2.292, 5.671)	0.000

**TABLE 7:** Multiple linear regression analysis of dentists’ stress status during the COVID-19 epidemic.

Variable	Stress (DASS-42 Score)	
	β (95%CI)	*p*
General health status	−0.366 (−4.824, −2.665)	0.000
Level of education	−0.155 (−3.702, −0.610)	0.006
Years of working	0.118 (0.088, 2.532)	0.036
Worried about occupational exposure/viral infection	0.146 (0.620, 3.871)	0.007
Overtime work during the outbreak	0.111 (0.027, 2.227)	0.045
Stress has nowhere to go, and the need for psychological counseling cannot be met	0.226 (2.246, 6.165)	0.000

### Results of Perceived Wellness Survey

As shown in Table 8, we found no significant difference in the mean of PWS between different demographic groups. Correlation analysis was carried out between the six PWS subscales and the depression, anxiety, and stress scores. The results showed a positive correlation between emotional and anxiety scores (*p* < 0.05). The social domain was negatively correlated with depression, anxiety, and stress (*p* < 0.05). The physical domain was negatively correlated with depression and stress (*p* < 0.05). Furthermore, the spiritual domain was positively associated with depression, anxiety, and stress (*p* < 0.05), as shown in Table 9.

**TABLE 8:** Perceived wellness survey (PWS) comparison with different demographic characteristics.

Demographic characteristics	Perceived wellness mean (SD)	*p*
**Gender**		
Male	134.35 (25.74)	0.094
Female	129.11 (21.47)	
**Age**		
21–30	129.48 (23.48)	0.436
31–40	131.98 (22.08)	
41–50	133.94 (18.54)	
≥51	120.13 (34.90)	
**Live alone**		
Yes	134.50 (29.82)	0.073
No	128.95 (18.81)	
**Marital status**		
Unmarried	128.75 (27.00)	0.325
Married	131.72 (20.43)	
**Fertility status**		
None	128.93 (25.37)	0.494
1 Child	131.73 (22.10)	
≥2 Children	133.50 (15.28)	
**Past medical history**		
Yes	138.10 (41.45)	0.132
No	130.06 (15.28)	
**Level of education**		
College or below	130.09 (24.42)	0.159
Undergraduate	128.98 (19.79)	
Postgraduate and above	133.91 (21.78)	
**Years of working**		
Under 5 years	129.22 (23.48)	0.696
6–10 Years	132.98 (22.65)	
More than 11 years	130.30 (22.36)	
**Professional title**		
Junior	128.94 (18.28)	0.343
Intermediate	133.98 (27.73)	
Senior	126.65 (27.36)	
**Medical institution**		
Public specialized hospitals	130.09 (24.42)	0.471
Pubic general hospitals	128.98 (19.79)	
Private Dental Clinics	133.91 (21.78)	

**TABLE 9:** Bivariate correlation analysis of PWS subscales with depression, anxiety, and stress.

	Physical		Emotional		Social		Psychological		Spiritual		Intellectual	
	*r*	*p*	*r*	*p*	*r*	*p*	*r*	*p*	*r*	*p*	*r*	*p*
Depressed	−0.060	0.337	0.113	0.070	−0.192**	0.002	−0.133*	0.033	0.198**	0.001	−0.100	0.109
Anxious	0.002	0.973	0.142*	0.023	−0.126*	0.044	−0.045	0.477	0.202**	0.001	−0.022	0.730
Stress	−0.055	0.382	0.083	0.185	−0.207**	0.001	−0.124*	0.047	0.125*	0.045	−0.101	0.107

*p < 0.05; **p < 0.01.

## Discussion

COVID-19 affects physical health and has a significant psychological impact globally (18). The following conclusions were drawn from our research: first, there were 33.2% for depression, 34.4% for anxiety, and 34.4% for stress among the 256 dentists in Shaanxi Province. Second, after analysis, we found many influencing factors that could lead to depression, anxiety, and stress. Third, there was a correlation between perceived health findings and depression, anxiety, and stress.

From previous research, it has been found that COVID-19 has a profound impact on the dental industry (19). The unique work environment puts dentists at a high risk of exposure and infection (20). In addition, isolation, unemployment, and fear of infecting family members can influence dentists’ mental health status during the COVID-19 pandemic (21). In our study, 95% of subjects were concerned about occupational exposure or viral infection, and 33% were worried about infection from colleagues. This finding is similar to many studies in which more than 80% of dentists fear infection in diagnosis and treatment by Zeina Nasser et al. (22). Additionally, Consolo et al. (23) found that nearly 85% of dentists were concerned about infection in daily clinical practice. In our univariate linear regression analysis of the factors affecting depression, anxiety, and stress status, we found that “fear of occupational exposure/viral infection” was positively associated with depression, anxiety, and stress (*p* < 0.05). Some dentists said they felt anxiety about treating patients with cough and fever (24). In our survey, 23.1% of dentists participated in outbreak-related operations (such as supporting nucleic acid sampling, pre-testing, triage, supporting fever clinics, vaccination, etc.), which was an influential factor of stress (*p* < 0.05). Furthermore, among our study subjects, 65.2% of the dentists were married, and 68.8% lived with their family members. When faced with a high-risk working environment and concerns about their family members, depression and anxiety were more likely to occur or aggravate these concerns, just as we observed. These results are the same as during the 2003 SARS outbreak. Looking back at the SARS epidemic in 2003, many studies found that medical staff had anxiety, depression, and stress symptoms at that time (25). They were afraid of occupational exposure at work, fearful of bringing the disease to family members and friends, and even reluctant to work or considered quitting the job (26). Collectively, these all can have long-term psychological effects (27).

Due to the requirements of epidemic prevention and control during the epidemic, some medical institutions have had to suspend treatment, especially private clinics. This puts a strain on dentists working in private practices. During the outbreak in India, 60.4% of oral clinics were closed for 2–3 months, 32.6% for 1 month, and 3.3% did not re-open after closure (28). Due to the closure of private oral clinics, 72.5% were worried about economic losses during the COVID-19 outbreak, and 37% of Vietnamese dental surgeons even though they were at a low quality of life level, which caused great stress and anxiety for dental practitioners working in private clinics (29). Our study found that dentists working in government hospitals account for more depression, anxiety, and stress (45.9, 44.2, and 44.3%). However, this result is different from previous studies. In previous studies (30), dentists working in clinics were significantly more stressed during the pandemic than dentists in government hospitals. These findings may be related to the closure of dental clinics during the epidemic and the inability to guarantee the dentist’s income (31). We analyzed the possible reasons: (1) When private clinics are closed, government hospitals take on more patients, dentists’ workload increases, working hours are longer, and the risk of infection increases, so they are more likely to produce psychological problems, such as anxiety and stress. (2) Among the study subjects, more doctors were working in government hospitals, so there may be a deviation in the sample results. Interestingly, our study found that education level was inversely associated with stress status, which was similar to Chen et al. (32).

Our study found that the impact of the pandemic on sleep was positively correlated with depression, anxiety, and stress status. There was an increasing number of evidence of a bidirectional relationship between psychosomatic conditions and insomnia, and this relationship may increase with stress (33). The study by Magdalena et al. also confirmed this relationship (34). Additionally, Lee et al. (35) found that sleep quality was inversely correlated with anxiety. Furthermore, many researchers have pointed out the effects of stress and anxiety on sleep (36).

A highlight of this study is that we used the PWS to evaluate subjects’ mental health status in six dimensions: physical, emotional, social, psychological, spiritual, and intellectual. This survey is not commonly used in relevant studies in China. Epidemiological researchers have concluded that self-health perception is one of the most powerful predictors of their future health status (17). The perceived wellness model was built on system theory and health orientation. According to systems theory, each part of a system was a sub-element of a more extensive and separate system with its sub-elements, and the elements were interrelated (37). In the correlation analysis of PWS subscales and depression, anxiety, and stress status, we found that (1) Physical and intellectual domains were inversely associated with depression and stress status; (2) Social domain was inversely associated with depression, anxiety, and stress status; (3) Spiritual domain was positively correlated with depression, anxiety, and stress status. There were correlations between the various dimensions of PWS and between each dimension and depression, anxiety, and stress level, indicating that multiple factors influenced an individual’s physical and mental health status. Another noteworthy aspect of our study was that general health status was negatively correlated with depression, anxiety, and stress. Other than that, the general health status is also related to perceived wellness. Previous studies (38) have shown that the degree of physical health was inversely correlated with the incidence of mental illness. Good perceived health and high levels of physical health were positively correlated and negatively correlated with physical and mental illnesses (39).

A comparative analysis of changes in mental health status in different periods cannot be conducted from our study since our investigation was conducted over a brief period, did not involve dynamic and continuous monitoring, and lacked records of dentists’ mental health status in different periods. Notably, the following psychological interventions are proposed in the “Guiding Principles for Emergency Psychological Crisis Intervention in the Novel Coronavirus Pneumonia Epidemic” issued by the National Health Commission: (1) Reasonable scheduling to ensure adequate sleep and diet; (2) Communicate more with family and friends; (3) Moderate exercise is beneficial to relieve the state of high mental tension, eliminate tension, release psychological pressure, promote deep sleep, and ensure medical staff’s physical and psychological health.

To sum up, the COVID-19 epidemic has indeed affected the psychology of dentists to varying degrees. Physical health, years of working, income, and working conditions during the epidemic were all related to the mental health of dentists. Therefore, it is very important to pay attention to the mental health of dentists and establish an effective psychological intervention system to reduce the psychological damage caused by the epidemic.

## Data Availability Statement

The raw data supporting the conclusions of this article will be made available by the authors, without undue reservation.

## Ethics Statement

The studies involving human participants were reviewed and approved by the Medical Ethics Committee of Xi’an Jiaotong University, Hospital of Stomatology, No. xjkqll (2022)NO.001. The patients/participants provided their written informed consent to participate in this study.

## Author Contributions

JL analyzed the data and wrote the manuscript. CC modified the manuscript. All authors worked together to design the study proposal and questionnaires and contributed to the article and approved the submitted version.

## Conflict of Interest

The authors declare that the research was conducted in the absence of any commercial or financial relationships that could be construed as a potential conflict of interest.

## Publisher’s Note

All claims expressed in this article are solely those of the authors and do not necessarily represent those of their affiliated organizations, or those of the publisher, the editors and the reviewers. Any product that may be evaluated in this article, or claim that may be made by its manufacturer, is not guaranteed or endorsed by the publisher.

## Footnotes

1
http://www.wjx.com
